# Cardiometabolic risk and vascular changes in rheumatic diseases

**DOI:** 10.1007/s00296-026-06129-w

**Published:** 2026-05-19

**Authors:** Yuliya Fedorchenko, Umida Khojakulova, Bekzhan A. Permenov, Mykhailo Fedorchenko, Ahmet Usen

**Affiliations:** 1 Department of Pathophysiology, Ivano-Frankivsk National Medical University, Ivano-Frankivsk, Ukraine; 2 Department of Emergency Medicine and Nursing, South Kazakhstan Medical Academy, Shymkent, Kazakhstan; 3 Department of Social Health Insurance and Public Health, South Kazakhstan Medical Academy, Shymkent, Kazakhstan; 4 Department of Cardiac Surgery Anesthesiology and Intensive Care, Heart Center Shymkent, Shymkent, Kazakhstan; 5 Department of Internal Medicine, Khoja Akhmet Yassawi International Kazakh-Turkish University, Turkistan, Kazakhstan; 6 Department of Therapy, Family and Emergency Medicine of the Faculty of Medicine, Ivano-Frankivsk National Medical University, Ivano-Frankivsk, Ukraine; 7 Department of Physical Medicine and Rehabilitation, Faculty of Medicine, Medipol University, Istanbul, Türkiye

**Keywords:** Rheumatic diseases, Atherosclerosis, Cardiovascular diseases, Endothelial dysfunction, Inflammation

## Abstract

Rheumatic diseases (RDs) are chronic immune-mediated disorders associated with disproportionately increased cardiovascular morbidity and mortality. Accelerated atherogenesis in these diseases is driven by persistent systemic inflammation, autoantibody-mediated endothelial injury, oxidative stress, and dysregulated lipid metabolism, resulting in premature vascular remodeling manifested by increased carotid intima–media thickness, arterial stiffness, impaired flow-mediated dilation, and coronary artery calcification. This review synthesizes evidence regarding subclinical atherosclerosis and cardiometabolic risk across common RDs. In rheumatoid arthritis and systemic lupus erythematosus, vascular alterations correlate with inflammatory burden, disease duration, autoantibody profiles, renal involvement, and glucocorticoid exposure. Emerging biomarkers—including apolipoprotein B48, FIB-4 index, asymmetric dimethylarginine, and adhesion molecules—provide incremental prognostic value beyond traditional lipid parameters. Advanced imaging modalities, such as ^18F-sodium fluoride PET/CT and vascular elastography, enhance early detection of arterial calcification and stiffness. Growing evidence in primary Sjögren syndrome, Behçet disease, systemic sclerosis, and ankylosing spondylitis similarly confirms increased subclinical atherosclerosis and endothelial dysfunction. Importantly, tight disease control and targeted immunomodulatory therapies—including methotrexate, biologic agents, antimalarials, and cytokine-directed treatments—are associated with improved vascular and metabolic profiles and attenuation of disease progression. Subclinical atherosclerosis represents a critical interface between autoimmunity and cardiovascular disease in RDs. Early vascular assessment integrated with disease-specific and metabolic risk stratification is essential to implement precision-based cardiovascular prevention in this high-risk population.

## Introduction

Rheumatic diseases (RDs), such as rheumatoid arthritis (RA), systemic lupus erythematosus (SLE), systemic sclerosis (SSc), primary Sjögren syndrome (pSS), and Behçet disease (BD), are chronic immune-mediated disorders characterized by persistent systemic inflammation and multi-organ involvement [1]. Patients with RDs are at increased risk of cardiovascular disease (CVD) [2]. The risk of myocardial infarction, stroke, and other atherothrombotic events is high in RDs [3–5].

Chronic systemic inflammation, autoantibody-mediated vascular injury, endothelial dysfunction, oxidative stress, and dysregulated lipid metabolism contribute to the heightened cardiovascular risk in RDs [6–8]. These pathological processes contribute to premature vascular alterations manifested with increased carotid intima–media thickness, arterial stiffness, and endothelial impairment [9]. Subclinical atherosclerosis has been recognized as a pivotal determinant of cardiovascular risk in RA and SLE [10, 11].

The interplay between disease-specific factors, such as autoantibody profiles, disease duration, and immunomodulatory therapy, and established cardiovascular risk factors has not been fully elucidated in the context of RDs. Addressing these knowledge gaps is essential for tailoring management and rehabilitation strategies in these diseases.

The current review aims to evaluate subclinical atherosclerosis and associated risk factors in RDs to provide insights into the links between (auto)immune disorders and vascular pathology and to prioritize early diagnosis and risk reduction strategies.

### Search strategy

Literature searches were conducted via Medline/PubMed, Scopus, and Web of Science to identify relevant studies published up to 2026. Previously published recommendations on comprehensive search strategies were followed [12]. Search terms included combinations of “autoimmune rheumatic diseases,” “rheumatoid arthritis,” “systemic lupus erythematosus,” “systemic sclerosis,” “primary Sjögren’s syndrome,” “Behçet’s disease,” “subclinical atherosclerosis,” “cardiovascular risk,” and “vascular imaging.” English original research articles and reviews were retrieved and analysed. Reference lists of the retrieved articles were manually screened to identify additional relevant studies. The articles were selected based on their relevance to the assessment of subclinical atherosclerosis and cardiometabolic risk factors in RDs, with preference given to recent observational, cohort, and clinical studies.

## Rheumatoid arthritis

RA accelerates atherogenesis via persistent systemic immune inflammation, autoantibody-mediated vascular injury, and endothelial dysfunction [13], conferring a 55% higher risk of symptomatic cardiovascular events compared with the general population (RR 1.55, 95% CI 1.18–2.02) [14]. Persistent rheumatoid inflammation triggers endothelial dysfunction, adipokine dysregulation, and insulin resistance, contributing to early vascular remodeling and subclinical atherosclerosis [15, 16]. Comparative analyses revealed elevated HOMA-IR, correlated with DAS28, hsCRP, TNF-α, resistin, and leptin, with significantly greater carotid intima–media thickness (CIMT) in RA compared with non-RA controls (p = 0.0062) [15]. Flow-mediated dilation (FMD) studies confirmed endothelial dysfunction in RA, with RA patients presenting with lower FMD (p < 0.001) and greater CIMT (p = 0.003) [16]. CRP, TNF-α, and IL-6 were strongly associated with vascular indices in RA, highlighting their predictive value for cardiovascular risk assessment [16].

Epicardial fat thickness (EFT) has emerged as a sensitive indicator of cardiometabolic risk in RA [17]. A case–control analysis demonstrated significantly increased EFT and diastolic dysfunction in RA (p < 0.05) [17]. Similarly, another cross-sectional study on 66 RA patients confirmed higher EFT in RA (p < 0.001) and revealed its association with RF, anti-CCP, ESR, and systolic blood pressure [18].

Vitamin D deficiency is prevalent in RA and independently associated with adverse metabolic and endothelial profiles [19]. In a study on 179 RA patients without established cardiovascular disease, 41% had 25(OH)D levels below 30 ng/mL and only 13% had this marker’s optimal levels (≥ 45 ng/mL) [19]. Higher 25(OH)D significantly and positively correlated with HDL and inversely with HOMA-IR, fibrinogen, E-selectin, and s-ICAM [19].

Population studies have confirmed the accelerated course of atherogenesis in RA [20, 21]. In a cohort study on 212 RA patients, cardiovascular treat-to-target strategies attenuated CIMT progression compared with standard therapy [20]. Metabolic syndrome amplified baseline CIMT and plaque frequency, with vascular improvements limited to patients without metabolic syndrome [20]. A carotid ultrasound study on 103 RA patients demonstrated a higher frequency of bilateral plaques and increased CIMT ≥ 0.9 mm in RA patients compared with non-RA controls [21].

Drug therapies, particularly with methotrexate and biologic therapies, confer significant cardiovascular benefits in RA [22, 23]. Methotrexate therapy is dose-dependently associated with lower CIMT compared with non-users [22]. A systematic review of studies on 195,416 RA patients confirmed that those on methotrexate therapy have reduced risk of cardiovascular events (RR 0.798, 95% CI 0.726–0.876) [24].

Biologic therapies improve lipid profiles and vascular outcomes in RA [23]. A systematic review demonstrated that biologics reduce triglycerides and LDL-C [23]. Consistent with these findings, a prospective study reported that anti-TNF and non-anti-TNF biologics increase total cholesterol, HDL-C, and apolipoprotein A1 at 3 months but reduce CIMT at 6 months in RA patients [25]. Six-month therapy with biologic DMARDs significantly decrease CIMT and anti-oxidized LDL antibody levels (p < 0.05) [26]. Timely anti-inflammatory therapy also improves HDL-mediated cholesterol efflux capacity independent of HDL-C levels [27]. However, not all antirheumatic therapies demonstrate consistent cardiovascular benefits, and for some agents, available data remain limited or inconclusive regarding their effects on cardiovascular outcomes [2].

The management of established cardiovascular risk factors significantly reduces subclinical atherosclerosis in RA. In fact, a 5-year randomized controlled trial of 320 RA patients demonstrated reduced CIMT progression and lower cardiovascular events (1.3% vs. 4.7%; p = 0.048) in a treat-to-target RA cohort [28].

Novel biomarkers, including apo B48 and FIB-4, identify RA patients at elevated cardiometabolic risk independent of traditional lipid measures or disease activity [29–31]. According to a cross-sectional study, RA patients had significantly higher apolipoprotein B48 levels than healthy controls (p < 0.001) [29]. Apo B48 correlated with triglycerides (r = 0.645, p < 0.001), and RA patients in the highest apo B48 tertile were more frequently seropositive [29]. FIB-4 measurements may identify patients with subclinical atherosclerosis and atherogenic lipid profiles [30]. In 326 RA patients, those with elevated FIB-4 presented with higher CIMT (p = 0.002) and anti-CCP levels (p = 0.02) [30].

Liver fibrosis has emerged as a potential contributor to the heightened cardiometabolic risk in RA [31]. In a study on 465 RA patients, 20% had FIB-4 scores indicating intermediate or high hepatic fibrosis risk, and FIB-4 correlated with SCORE2-estimated cardiovascular risk, insulin resistance, and metabolic syndrome [31].

Advanced imaging modalities, such as ^18F-sodium fluoride PET/CT, detect early vascular calcification more accurately than ^18F-FDG in RA, with patients demonstrating higher NaF uptake and greater CT calcium volume, indicating its utility as a subclinical atherosclerosis biomarker [32]. Biochemical markers such as asymmetric dimethylarginine (ADMA) and adhesion molecules, including ICAM-1, VCAM-1, PECAM-1, E-selectin, and P-selectin, are elevated in RA, linking inflammation to endothelial dysfunction and subclinical atherosclerosis [33, 34].

Tocilizumab therapy enhances cholesterol efflux capacity and demonstrates atheroprotective effects independent of BMI or body composition [35, 36]. In a 12-month clinical trial, tocilizumab reduced systemic inflammation and cholesterol levels [35]. Abatacept and adalimumab also improve HDL composition and function, decreasing inflammatory HDL proteins and increasing paraoxonase-1 activity [37]. Puerarin and doxycycline mitigate insulin resistance and CIMT increase in RA [38, 39]. In a 24-week trial, puerarin reduced CIMT and improved HOMA-IR, with a strong correlation between vascular and metabolic improvements [38], while methotrexate combined with doxycycline reduced inflammatory markers and disease activity more effectively than MTX alone [38, 39].

### Systemic lupus erythematosus

Subclinical atherosclerosis is prevalent in SLE [9]. Both established (age, HDL, triglycerides) and disease-specific risk factors (disease duration, SLEDAI, corticosteroid use) influence CIMT, while steroids and triglycerides contribute to carotid plaque frequency [9]. Increased CIMT, higher plaque frequency, and reduced flow-mediated dilation have been reported in SLE patients [40]. Endothelial dysfunction and increased arterial stiffness are characteristic features of SLE [41].

Established cardiovascular risk factors remain key determinants of atherosclerosis progression in SLE [42]. Although guidelines for cardiovascular risk management in rheumatic diseases are well established (EULAR recommendations) [43], real-world evidence regarding the implementation and effectiveness of primary prophylactic strategies remains limited. In a two-year study on 187 patients, coronary artery calcification (CAC) increased in parallel to total cholesterol and smoking [42]. The emergence of new carotid plaques was associated with systolic BP and disease duration along with many other factors [42]. Similarly, a five-year study on 99 SLE patients demonstrated that CAC progression was significantly associated with prior smoking (RR 1.69, 95% CI 1.19–2.4), longer disease duration (RR per year 1.03, 95% CI 1.01–1.04), and baseline CAC (RR 2.52, 95% CI 1.68–3.78) [44].

Subclinical atherosclerosis is particularly pronounced in SLE patients with renal involvement [44]. In a cohort of 100 lupus patients, nephritis was linked to reduced FMD and higher SLEDAI scores [45].

Obesity and metabolic derangements further exacerbate cardiovascular risk in lupus [46]. Obesity has been associated with higher lupus activity, increased organ damage, elevated inflammatory markers, dyslipidemia, and higher systolic BP [46]. In obese women with SLE, a 12-week low-calorie diet combined with Pilates and laser acupuncture improved lipid profiles, suggesting that multimodal strategies targeting inflammation and metabolism may enhance cardiometabolic health in SLE [47]. Conversely, sedentary SLE patients demonstrated blunted lipid and HDL responses to exercise compared with healthy controls [48].

Drug therapies, particularly antimalarial therapy, also influence cardiovascular outcomes in SLE [49]. A systematic review proved that hydroxychloroquine therapy exerts atheroprotective effects by lowering triglycerides, diastolic BP, and diabetes incidence (HR 0.39, 95% CI 0.17–0.88) [49]. Additionally, phase 2 trials of cenerimod in moderate-to-severe SLE demonstrated significant reductions in mSLEDAI-2 K scores, with dose-dependent lymphopenia and an acceptable safety profile [50, 51]. Type I interferon blockade via anifrolumab also improved cholesterol efflux capacity and restored endothelial function in SLE [52]. Emerging evidence suggests that glucagon-like peptide-1 receptor agonists (GLP-1RA) may exert beneficial cardiometabolic and anti-inflammatory effects, which could be relevant in patients with rheumatic diseases; however, their clinical impact remains incompletely understood and requires further investigation [53].

Markers of vascular calcification and subclinical atherosclerosis have been extensively investigated in SLE [54–56]. Higher organ damage and glucocorticoid use correlate with increased CAC in lupus [54]. Inflammatory pathways remain central to cardiovascular risk in SLE [57]. Chronic systemic inflammation correlates with CAC, while age and traditional metabolic risk factors modulate this association [57, 58].

### Sjögren syndrome

Primary Sjögren syndrome (pSS) is associated with subclinical atherosclerosis [59]. A systematic review of 19 studies (n = 1,625) demonstrates higher frequency of atherosclerotic plaques in pSS (OR = 1.9; 95% CI 1.32–2.74) [59]. Observational studies further corroborate these findings, highlighting early vascular impairment in the absence of overt structural changes [60, 61]. Oxidized LDL antibodies significantly correlated with pSS activity and SSA/Ro positivity in pSS [61]. A systematic review of observational studies reinforce the presence of early vascular abnormalities in pSS [62]. Notably, the prevalence of subclinical atherosclerosis in pSS has been comparable to that in type 2 diabetes mellitus [63].

Therapeutic strategies targeting immune modulation may impact vascular and metabolic pathways in pSS [64]. In a double-blind randomized controlled trial on 60 pSS patients, 12 weeks of low-dose interleukin-2 therapy in combination with standard therapy led to significant changes in acetyl-CoA, ascorbic acid, and glutathione, correlating with Treg cell variations in pSS (p < 0.05) [64].

### Behçet disease

BD is increasingly recognized as a nosological entity associated with endothelial dysfunction and subclinical atherosclerosis [65]. In a cohort of 34 BD patients and 28 controls, CIMT and common carotid artery stiffness were significantly higher in patients [66]. Shear wave elastography (SWE) further refines assessment, distinguishing active from inactive BD [67]. In 39 BD patients (19 active, 20 inactive) and 22 controls, active-stage patients showed elevated SWE-measured stiffness [67].

Markers of early vascular dysfunction also provide important prognostic information [68, 69]. Serum irisin, a myokine linked to endothelial health, was evaluated in BD patients alongside CIMT [68]. BD patients exhibited higher CIMT and lower irisin levels, independent of sex and BMI [68]. A pooled analysis of 36 case–control studies (1,103 BD patients, 832 controls) demonstrated significantly increased CIMT in BD (p < 0.0001; I^2^ = 86.9%), with heterogeneity partly explained by male sex, age, and azathioprine use [70]. Vascular BD patients had higher CIMT than non-vascular patients (p = 0.006), and carotid plaques were more prevalent in patients compared with controls (13.1% vs. 2.97%; p < 0.0001) [70].

### Systemic sclerosis

SSc confers an increased cardiovascular risk, primarily driven by endothelial dysfunction, vascular injury, and accelerated atherogenesis [71]. A systematic review of 43 studies demonstrated that patients with SSc exhibited significantly elevated levels of ICAM-1, VCAM-1, PECAM-1, E-selectin, and P-selectin [71]. Vascular dysfunction in SSc is further characterized by alterations in angiogenic signaling [72]. A systematic review of 42 studies reported significantly elevated vascular endothelial growth factor (VEGF) in SSc patients compared with controls; VEGF concentrations were higher in diffuse compared with localized disease, and in patients with pulmonary hypertension [72]. Collectively, these data highlight the utility of circulating adhesion molecules and VEGF as complementary markers to monitor endothelial dysfunction, disease progression, and cardiovascular risk in patients with SSc.

### Ankylosing spondylitis

Patients with AS exhibit an increased risk of subclinical atherosclerosis, highlighting the importance of early cardiovascular screening and intervention [73]. A systematic review of 35 studies including 1,535 AS patients and 1,347 controls demonstrated significantly elevated CIMT and pulse wave velocity, accompanied by reduced FMD, confirming early arterial dysfunction in AS [73]. Cardiovascular complications are also prevalent in axial spondyloarthritis, with a systematic review of 123 studies revealing that established cardiovascular risk factors are closely associated with persistent disease activity, emphasizing the need for proactive cardiovascular risk management alongside tight disease control [74]. A systematic review found that HDL-C was significantly lower in AS patients (p < 0.001) [75]. Consistent with these findings, a cohort study demonstrated higher CIMT and elevated atherogenic index in AS [76].

## Conclusion

**Fig. 1:**
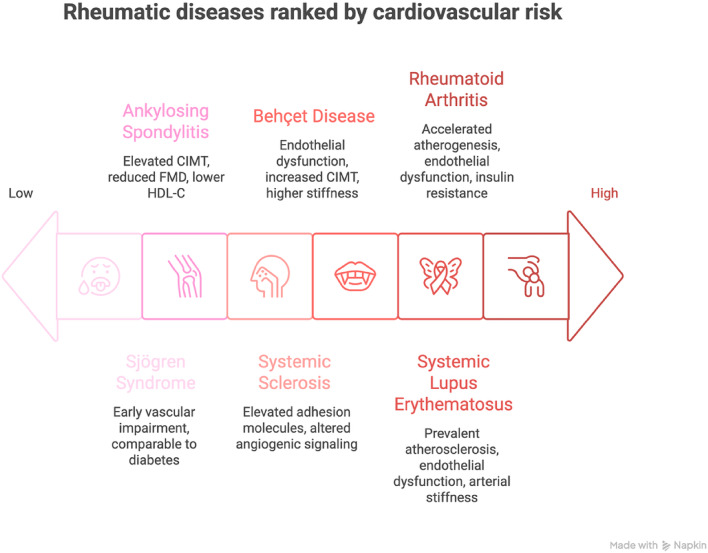
Cardiovascular Risk in Rheumatic Diseases

Autoimmune RDs, including RA, SLE, pSS, BD, SSc, and AS, are intrinsically associated with subclinical atherosclerosis and early vascular dysfunction. Schematic representation of RDs ranked according to comparative cardiovascular risk and predominant vascular alterations is depicted in Fig. 1. This scheme is in line with a previously discussed concept of atherogenesis differing in high and low-grade inflammatory RDs [77].

Persistent systemic inflammation, endothelial injury, immune dysregulation, and metabolic disturbances converge to drive arterial stiffening, endothelial impairment, and atherogenic remodeling, often preceding overt cardiovascular events in RDs. Advanced imaging, vascular stiffness indices, and emerging biomarkers (e.g., epicardial fat thickness, apoB48, irisin, FIB-4) enable sensitive detection and risk stratification. Targeted pharmacologic and immunometabolic interventions improve vascular function, underscoring the necessity of integrated cardiovascular risk management alongside disease-specific therapy. Early identification and proactive management of subclinical vascular disease are pivotal to reducing cardiovascular morbidity and optimizing long-term outcomes in these high-risk populations.

## References

[1] Navid F, Colbert RA (2017) Causes and consequences of endoplasmic reticulum stress in rheumatic disease. Nat Rev Rheumatol 13(1):25–40. 10.1038/nrrheum.2016.192 27904144 10.1038/nrrheum.2016.192

[2] Nurmohamed MT, Heslinga M, Kitas GD (2015) Cardiovascular comorbidity in rheumatic diseases. Nat Rev Rheumatol 11(12):693–704. 10.1038/nrrheum.2015.112 26282082 10.1038/nrrheum.2015.112

[3] Fedorchenko Y, Suigenbayev D, Sagtaganov Z, Imanbayeva N, Mahmudzoda K (2025) Myocardial infarction in rheumatic diseases. Rheumatol Int 45(12):270. 10.1007/s00296-025-06032-w 41212291 10.1007/s00296-025-06032-w

[4] Wiseman SJ, Ralston SH, Wardlaw JM (2016) Cerebrovascular disease in rheumatic diseases: a systematic review and meta-analysis. Stroke 47(4):943–950. 10.1161/STROKEAHA.115.012052 26917565 10.1161/STROKEAHA.115.012052

[5] Gasparyan AY (2023) Cardiovascular manifestations and comorbidities in rheumatic diseases: perspectives on timely diagnosis, prevention, and treatment. Clin Rheumatol 42(10):2531–2533. 10.1007/s10067-023-06762-x 37698746 10.1007/s10067-023-06762-x

[6] Zimba O, Gasparyan AY (2023) Cardiovascular issues in rheumatic diseases. Clin Rheumatol 42(10):2535–2539. 10.1007/s10067-023-06656-y 37269421 10.1007/s10067-023-06656-y

[7] Drosos AA, Venetsanopoulou AA, Pelechas E, Voulgari PV (2024) Exploring cardiovascular risk factors and atherosclerosis in rheumatoid arthritis. Eur J Intern Med 128:1–9. 10.1016/j.ejim.2024.07.016 39048336 10.1016/j.ejim.2024.07.016

[8] Husaini ASA, Fathima A, Halawa D, Aakel N, Erre GL, Giordo R, Zayed H, Pintus G (2025) Exploring endothelial dysfunction in major rheumatic diseases: current trends and future directions. J Mol Med (Berl) 103(6):635–649. 10.1007/s00109-025-02539-8 40229608 10.1007/s00109-025-02539-8 PMC12141126

[9] Tyrrell PN, Beyene J, Feldman BM, McCrindle BW, Silverman ED, Bradley TJ (2010) Rheumatic disease and carotid intima-media thickness: a systematic review and meta-analysis. Arterioscler Thromb Vasc Biol 30(5):1014–1026. 10.1161/ATVBAHA.109.198424 20150560 10.1161/ATVBAHA.109.198424

[10] Ruscitti P, Cipriani P, Liakouli V, Iacono D, Pantano I, Margiotta DPE, Navarini L, Destro Castaniti GM, Maruotti N, Di Scala G, Picciariello L, Caso F, Bongiovanni S, Grembiale RD, Atzeni F, Scarpa R, Perosa F, Emmi G, Cantatore FP, Guggino G, Afeltra A, Ciccia F, Giacomelli R (2019) Subclinical and clinical atherosclerosis in rheumatoid arthritis: results from the 3-year, multicentre, prospective, observational GIRRCS (Gruppo Italiano di Ricerca in Reumatologia Clinica e Sperimentale) study. Arthritis Res Ther 21(1):204. 10.1186/s13075-019-1975-y 31481105 10.1186/s13075-019-1975-y PMC6724256

[11] Tobin R, Patel N, Tobb K, Weber B, Mehta PK, Isiadinso I (2023) Atherosclerosis in Systemic Lupus Erythematosus. Curr Atheroscler Rep 25(11):819–827. 10.1007/s11883-023-01149-4 37768411 10.1007/s11883-023-01149-4

[12] Gasparyan AY, Ayvazyan L, Blackmore H, Kitas GD (2011) Writing a narrative biomedical review: considerations for authors, peer reviewers, and editors. Rheumatol Int 31:1409. 10.1007/s00296-011-1999-3 21800117 10.1007/s00296-011-1999-3

[13] Raj R, Thomas S, Gorantla V (2022) Accelerated atherosclerosis in rheumatoid arthritis: a systematic review. F1000Res 11:466. 10.12688/f1000research.112921.2 36249997 10.12688/f1000research.112921.1 PMC9551388

[14] Restivo V, Candiloro S, Daidone M, Norrito R, Cataldi M, Minutolo G, Caracci F, Fasano S, Ciccia F, Casuccio A, Tuttolomondo A (2022) Systematic review and meta-analysis of cardiovascular risk in rheumatological disease: Symptomatic and non-symptomatic events in rheumatoid arthritis and systemic lupus erythematosus. Autoimmun Rev 21(1):102925. 10.1016/j.autrev.2021.102925 34454117 10.1016/j.autrev.2021.102925

[15] Guin A, Sinhamahapatra P, Misra S, Choudhury Mazumder SR, Chatterjee S, Ghosh A (2019) Incidence and effect of insulin resistance on progression of atherosclerosis in rheumatoid arthritis patients of long disease duration. Biomed J 42(6):394–402. 10.1016/j.bj.2019.01.007 31948603 10.1016/j.bj.2019.01.007 PMC6962725

[16] Verma I, Syngle A, Krishan P (2017) Predictors of endothelial dysfunction and atherosclerosis in rheumatoid arthritis in Indian population. Indian Heart J 69(2):200–206. 10.1016/j.ihj.2016.10.013 28460767 10.1016/j.ihj.2016.10.013 PMC5414984

[17] Saha S, Singh R, Mir IA, Bansal N, Singh PK, Nadeem M (2022) Epicardial Fat Thickness: A Cardiometabolic Risk Marker in Rheumatoid Arthritis. Cureus 14(1):e21397. 10.7759/cureus.21397 35198304 10.7759/cureus.21397 PMC8855100

[18] Delkash P, Bayat B, Omidi F (2024) Epicardial fat thickness in rheumatoid arthritis: Insights from echocardiographic analysis and autoimmune correlations. Int J Rheum Dis 27(8):e15272. 10.1111/1756-185X.15272 39152621 10.1111/1756-185X.15272

[19] Haque UJ, Bathon JM, Giles JT (2012) Association of vitamin D with cardiometabolic risk factors in rheumatoid arthritis. Arthritis Care Res (Hoboken) 64(10):1497–1504. 10.1002/acr.21715 22555877 10.1002/acr.21715 PMC3462271

[20] Burggraaf B, van Breukelen-van der Stoep DF, de Vries MA, Klop B, van Zeben J, van de Geijn GM, van der Meulen N, Birnie E, Prinzen L, Castro Cabezas M (2018) Progression of subclinical atherosclerosis in subjects with rheumatoid arthritis and the metabolic syndrome. Atherosclerosis 271:84–91. 10.1016/j.atherosclerosis.2018.02.019 29482038 10.1016/j.atherosclerosis.2018.02.019

[21] Wah-Suarez MI, Galarza-Delgado DA, Azpiri-Lopez JR, Colunga-Pedraza IJ, Abundis-Marquez EE, Davila-Jimenez JA, Guillen-Gutierrez CY, Elizondo-Riojas G (2019) Carotid ultrasound findings in rheumatoid arthritis and control subjects: A case-control study. Int J Rheum Dis 22(1):25–31. 10.1111/1756-185X.13377 30168277 10.1111/1756-185X.13377

[22] Kim HJ, Kim MJ, Lee CK, Hong YH (2015) Effects of Methotrexate on Carotid Intima-media Thickness in Patients with Rheumatoid Arthritis. J Korean Med Sci 30(11):1589–1596. 10.3346/jkms.2015.30.11.1589 26539002 10.3346/jkms.2015.30.11.1589 PMC4630474

[23] Jia X, Yang Z, Li J, Mei Z, Jia L, Yan C (2024) The impact of biologic agents on cardiovascular risk factors in patients with rheumatoid arthritis: A meta analysis. PLoS ONE 19(8):e0306513. 10.1371/journal.pone.0306513 39208032 10.1371/journal.pone.0306513 PMC11361434

[24] Sun KJ, Liu LL, Hu JH, Chen YY, Xu DY (2021) Methotrexate can prevent cardiovascular events in patients with rheumatoid arthritis: an updated meta-analysis. Medicine (Baltimore) 100(7):e24579. 10.1097/MD.0000000000024579 33607787 10.1097/MD.0000000000024579 PMC7899830

[25] Papamichail GV, Markatseli TE, Georgiadis AN, Xydis VG, Milionis H, Drosos AA, Voulgari PV (2022) The effects of biologic agents on cardiovascular risk factors and atherosclerosis in rheumatoid arthritis patients: a prospective observational study. Heart Vessels 37(12):2128–2136. 10.1007/s00380-022-02114-y 35739432 10.1007/s00380-022-02114-y

[26] Papamichail GV, Georgiadis AN, Tellis CC, Rapti I, Markatseli TE, Xydis VG, Tselepis AD, Drosos AA, Voulgari PV (2024) Antibodies against oxidized LDL and atherosclerosis in rheumatoid arthritis patients treated with biological agents: a prospective controlled study. Clin Rheumatol 43(1):481–488. 10.1007/s10067-023-06744-z 37642764 10.1007/s10067-023-06744-z

[27] Xie B, He J, Liu Y, Liu T, Liu C (2021) A meta-analysis of HDL cholesterol efflux capacity and concentration in patients with rheumatoid arthritis. Lipids Health Dis 20(1):18. 10.1186/s12944-021-01444-6 33612101 10.1186/s12944-021-01444-6 PMC7897392

[28] Burggraaf B, van Breukelen-van der Stoep DF, de Vries MA, Klop B, Liem AH, van de Geijn GM, van der Meulen N, Birnie E, van der Zwan EM, van Zeben J, Castro Cabezas M (2019) Effect of a treat-to-target intervention of cardiovascular risk factors on subclinical and clinical atherosclerosis in rheumatoid arthritis: a randomised clinical trial. Ann Rheum Dis 78(3):335–341. 10.1136/annrheumdis-2018-214075 30610067 10.1136/annrheumdis-2018-214075

[29] Burggraaf B, van Breukelen-van der Stoep DF, van Zeben J, van der Meulen N, van de Geijn GM, Liem A, Valdivielso P, Rioja Villodres J, Ramírez-Bollero J, van der Zwan E, Castro Cabezas M (2018) Evidence for increased chylomicron remnants in rheumatoid arthritis. Eur J Clin Invest. 10.1111/eci.12873 29231984 10.1111/eci.12873

[30] Saidi AN, Theel WB, Burggraaf B, van der Lelij AJ, Grobbee DE, van Zeben JD, van der Zwan-van Beek E, Rauh SP, Cabezas MC (2025) Metabolic dysfunction-associated steatotic liver disease and cardiovascular risk factors in rheumatoid arthritis. Clin Rheumatol 44(4):1485–1492. 10.1007/s10067-025-07364-5 39962010 10.1007/s10067-025-07364-5 PMC11993437

[31] Ferraz-Amaro I, Heras-Recuero E, de Vera-González A, González-Delgado A, Romo-Cordero A, Quevedo-Rodríguez A, Quevedo-Abeledo JC, Largo R, González-Gay MÁ (2025) The Fibrosis-4 Index (FIB-4) correlates with cardiovascular risk and insulin resistance in patients with rheumatoid arthritis. Rheumatology (Oxford) 64(9):5065–5073. 10.1093/rheumatology/keaf263 40397115 10.1093/rheumatology/keaf263

[32] Seraj SM, Raynor WY, Revheim ME, Al-Zaghal A, Zadeh MZ, Arani LS, Rojulpote C, Werner TJ, Gerke O, Høilund-Carlsen PF, Baker JF, Alavi A, Hunt SJ (2020) Assessing the feasibility of NaF-PET/CT versus FDG-PET/CT to detect abdominal aortic calcification or inflammation in rheumatoid arthritis patients. Ann Nucl Med 34(6):424–431. 10.1007/s12149-020-01463-w 32277422 10.1007/s12149-020-01463-w

[33] Zafari P, Zarifian A, Alizadeh-Navaei R, Taghadosi M, Rafiei A, Samimi Z, Niksolat F (2020) Asymmetric and symmetric dimethylarginine concentration as an indicator of cardiovascular diseases in rheumatoid arthritis patients: a systematic review and meta-analysis of case-control studies. Clin Rheumatol 39(1):127–134. 10.1007/s10067-019-04713-z 31376089 10.1007/s10067-019-04713-z

[34] Mangoni AA, Zinellu A (2024) A systematic review and meta-analysis of circulating adhesion molecules in rheumatoid arthritis. Inflamm Res 73(3):305–327. 10.1007/s00011-023-01837-6 38240792 10.1007/s00011-023-01837-6 PMC10894129

[35] Ferraz-Amaro I, Hernández-Hernández MV, Tejera-Segura B, Delgado-Frías E, Macía-Díaz M, Machado JD, Diaz-González F (2019) Effect of IL-6 receptor blockade on Proprotein Convertase Subtilisin/Kexin Type-9 and cholesterol efflux capacity in Rheumatoid Arthritis patients. Horm Metab Res 51(3):200–209. 10.1055/a-0833-4627 30695794 10.1055/a-0833-4627

[36] Hoffman E, Rahat MA, Feld J, Elias M, Rosner I, Kaly L, Lavie I, Gazitt T, Zisman D (2019) Effects of Tocilizumab, an anti-Interleukin-6 receptor antibody, on serum lipid and adipokine levels in patients with rheumatoid arthritis. Int J Mol Sci 20(18):4633. 10.3390/ijms20184633 31540528 10.3390/ijms20184633 PMC6770905

[37] Charles-Schoeman C, Gugiu GB, Ge H, Shahbazian A, Lee YY, Wang X, Furst DE, Ranganath VK, Maldonado M, Lee T, Reddy ST (2018) Remodeling of the HDL proteome with treatment response to abatacept or adalimumab in the AMPLE trial of patients with rheumatoid arthritis. Atherosclerosis 275:107–114. 10.1016/j.atherosclerosis.2018.04.003 29886354 10.1016/j.atherosclerosis.2018.04.003 PMC6113060

[38] Yang M, Luo Y, Liu T, Zhong X, Yan J, Huang Q, Tao J, He Q, Guo M, Hu Y (2018) The effect of Puerarin on carotid intima-media thickness in patients with active rheumatoid arthritis: ARandomized controlled trial. Clin Ther 40(10):1752-1764.e1. 10.1016/j.clinthera.2018.08.014 30245282 10.1016/j.clinthera.2018.08.014

[39] Ibrahem EM, El-Gendi SS, Mahmoud AA, Abdel-Aal SM, El Nouby FH, El-Deen Mohammed HS (2021) Predictors of cardiovascular affection in patients with active rheumatoid arthritis: secondary analysis of a randomized controlled trial. Curr Rheumatol Rev 17(2):258–266. 10.2174/1573397116666201113090145 33185166 10.2174/1573397116666201113090145

[40] Henrot P, Foret J, Barnetche T, Lazaro E, Duffau P, Seneschal J, Schaeverbeke T, Truchetet ME, Richez C (2018) Assessment of subclinical atherosclerosis in systemic lupus erythematosus: a systematic review and meta-analysis. Joint Bone Spine 85(2):155–163. 10.1016/j.jbspin.2017.12.009 29288864 10.1016/j.jbspin.2017.12.009

[41] Mendoza-Pinto C, Rojas-Villarraga A, Molano-González N, García-Carrasco M, Munguía-Realpozo P, Etchegaray-Morales I, Morales-Sánchez H, Berra-Romani R, Cervera R (2020) Endothelial dysfunction and arterial stiffness in patients with systemic lupus erythematosus: a systematic review and meta-analysis. Atherosclerosis 297:55–63. 10.1016/j.atherosclerosis.2020.01.028 32078830 10.1016/j.atherosclerosis.2020.01.028

[42] Kiani AN, Post WS, Magder LS, Petri M (2011) Predictors of progression in atherosclerosis over 2 years in systemic lupus erythematosus. Rheumatology (Oxford) 50(11):2071–2079. 10.1093/rheumatology/ker285 21875880 10.1093/rheumatology/ker285 PMC3247795

[43] Drosos GC, Vedder D, Houben E et al (2022) EULAR recommendations for cardiovascular risk management in rheumatic and musculoskeletal diseases, including systemic lupus erythematosus and antiphospholipid syndrome. Ann Rheum Dis 81(6):768–779. 10.1136/annrheumdis-2021-221733 35110331 10.1136/annrheumdis-2021-221733

[44] Zinglersen L, Zinglersen AH, Myhr KA, Hermansen ML, Kofoed KF, Fuchs A, Diederichsen LP, Jacobsen S (2025) Coronary artery calcification progression and renal involvement in patients with systemic lupus erythematosus: a longitudinal cohort study. Rheumatol Int 45(1):26. 10.1007/s00296-025-05785-8 39804493 10.1007/s00296-025-05785-8 PMC11729070

[45] Sharma SK, Rathi M, Sahoo S, Prakash M, Dhir V, Singh S (2016) Assessment of premature atherosclerosis in systemic lupus erythematosus patients with and without nephritis. Lupus 25(5):525–531. 10.1177/0961203315622822 26678442 10.1177/0961203315622822

[46] Correa-Rodríguez M, Pocovi-Gerardino G, Callejas Rubio JL, Ríos Fernández R, Martín Amada M, Cruz Caparrós M, Ortego-Centeno N, Rueda-Medina B (2020) The impact of obesity on disease activity, damage accrual, inflammation markers and cardiovascular risk factors in systemic lupus erythematosus. Panminerva Med 62(2):75–82. 10.23736/S0031-0808.19.03748-0 32515571 10.23736/S0031-0808.19.03748-0

[47] Ismail AMA, Saad AE, Abd-Elrahman NAF, Elfahl AMA (2023) Response of lipid profile to laser acupuncture along with diet and pilates exercise in obese women with systemic lupus erythematosus: a randomized controlled trial. J Acupunct Meridian Stud 16(4):152–158. 10.51507/j.jams.2023.16.4.152 37609770 10.51507/j.jams.2023.16.4.152

[48] Benatti FB, Miossi R, Passareli M, Nakandakare ER, Perandini L, Lima FR, Roschel H, Borba E, Bonfá E, Gualano B, de Sá Pinto AL (2015) The effects of exercise on lipid profile in systemic lupus erythematosus and healthy individuals: a randomized trial. Rheumatol Int 35(1):61–69. 10.1007/s00296-014-3081-4 24972700 10.1007/s00296-014-3081-4

[49] Munguía-Realpozo P, Mendoza-Pinto C, García-Carrasco M, Berra-Romani R, Sierra-Benito C, Méndez-Martínez S, Cervera R (2021) The impact of antimalarial agents on traditional and non-traditional subclinical atherosclerosis biomarkers in systemic lupus erythematosus: a systematic review and meta-analysis. Autoimmun Rev 20(9):102887. 10.1016/j.autrev.2021.102887 34237422 10.1016/j.autrev.2021.102887

[50] Askanase AD, D’Cruz D, Kalunian K, Merrill JT, Navarra SV, Cahuzac C, Cornelisse P, Murphy MJ, Strasser DS, Trokan L, Berkani O (2025) Cenerimod, a sphingosine-1-phosphate receptor modulator, versus placebo in patients with moderate-to-severe systemic lupus erythematosus (CARE): an international, double-blind, randomised, placebo-controlled, phase 2 trial. Lancet Rheumatol 7(1):e21–e32. 10.1016/S2665-9913(24)00246-7 39586304 10.1016/S2665-9913(24)00246-7

[51] Suffiotti M, Brazauskas P, Keller MP, Berkani O, Seifer G, Cornelisse P, Murphy MJ, Strasser DS (2025) Pharmacodynamics of the S1P_1_ receptor modulator cenerimod in a phase 2b randomised clinical trial in patients with moderate to severe SLE. Ann Rheum Dis 84(2):284–293. 10.1136/ard-2024-226547 39919901 10.1136/ard-2024-226547

[52] Casey KA, Smith MA, Sinibaldi D, Seto NL, Playford MP, Wang X, Carlucci PM, Wang L, Illei G, Yu B, Wang S, Remaley AT, Mehta NN, Kaplan MJ, White WI (2021) Modulation of cardiometabolic disease markers by type I interferon inhibition in systemic lupus erythematosus. Arthritis Rheumatol 73(3):459–471. 10.1002/art.41518 32909675 10.1002/art.41518 PMC11302498

[53] Bilgin E, Venerito V, Bogdanos DP (2025) Glucagon-Like Peptide-1 (GLP-1) receptor agonists in rheumatology: a review of current evidence and future directions. Autoimmun Rev 24(9):103864. 10.1016/j.autrev.2025.103864 40617296 10.1016/j.autrev.2025.103864

[54] Mendoza-Pinto C, Munguía-Realpzo P, García-Carrasco M, Godinez-Bolaños K, Rojas-Villarraga A, Morales-Etchegaray I, Ayón-Aguilar J, Méndez-Martínez S, Cervera R (2022) Asymptomatic coronary artery disease assessed by coronary computed tomography in patients with systemic lupus erythematosus: A systematic review and meta-analysis. Eur J Intern Med 100:102–109. 10.1016/j.ejim.2022.04.001 35410814 10.1016/j.ejim.2022.04.001

[55] Ocampo-Torres MC, Hernández-Molina G, Criales-Vera S, Sánchez-Guerrero J, Lara-Reyes P, Romero-Díaz J (2024) Coronary artery calcification progression in patients with systemic lupus erythematosus. J Rheumatol 51(10):991–996. 10.3899/jrheum.2024-0040 38950947 10.3899/jrheum.2024-0040

[56] Kiani AN, Aukrust P, Ueland T, Hollan I, Barr E, Magder LS, Petri M (2017) Serum osteoprotegrin (OPG) in subclinical atherosclerosis in systemic lupus erythematosus. Lupus 26(8):865–870. 10.1177/0961203316682101 27927880 10.1177/0961203316682101 PMC5397351

[57] Martínez-Ceballos MA, Sinning Rey JC, Alzate-Granados JP, Mendoza-Pinto C, García-Carrasco M, Montes-Zabala L, Vargas-Vergara D, Munguia-Realpozo P, Etchegaray-Morales I, Rojas-Villarraga A (2021) Coronary calcium in autoimmune diseases: a systematic literature review and meta-analysis. Atherosclerosis 335:68–76. 10.1016/j.atherosclerosis.2021.09.017 34592584 10.1016/j.atherosclerosis.2021.09.017

[58] Yang T, Qiu Y, Zhang Y, Hu W, Li M, Dai Y, Zhou Y, Yin Y (2024) The association of cardiovascular disease risk with coronary artery calcification and thoracic aortic dilation: a study in idiopathic inflammatory myopathies and systemic lupus erythematosus. Clin Rheumatol 43(10):3117–3125. 10.1007/s10067-024-07115-y 39186172 10.1007/s10067-024-07115-y

[59] Karakasis P, Patoulias D, Pamporis K, Stachteas P, Lefkou E, Bougioukas KI, Dimitroulas T, Fragakis N (2024) Risk of subclinical atherosclerosis in primary Sjogren’s syndrome: a systematic review and meta-analysis. Eur J Intern Med 122:93–101. 10.1016/j.ejim.2023.11.007 37977997 10.1016/j.ejim.2023.11.007

[60] Ozisler C, Kaplanoglu H (2019) Evaluation of subclinical atherosclerosis by ultrasound radiofrequency data technology in patients with primary Sjögren’s syndrome. Clin Rheumatol 38(3):709–717. 10.1007/s10067-018-4330-9 30334118 10.1007/s10067-018-4330-9

[61] Zehrfeld N, Abelmann M, Benz S, Zippel CL, Beider S, Kramer E, Seeliger T, Sogkas G, Gödecke V, Ahrenstorf G, Armbruster FP, Skripuletz T, Witte T, Derda AA, Sonnenschein K, Ernst D (2024) Primary Sjögren’s syndrome independently promotes premature subclinical atherosclerosis. RMD Open 10(2):e003559. 10.1136/rmdopen-2023-003559 38663882 10.1136/rmdopen-2023-003559 PMC11043759

[62] Yong WC, Sanguankeo A, Upala S (2019) Association between primary Sjogren’s syndrome, arterial stiffness, and subclinical atherosclerosis: a systematic review and meta-analysis. Clin Rheumatol 38(2):447–455. 10.1007/s10067-018-4265-1 30178172 10.1007/s10067-018-4265-1

[63] Zhang Y, Luo Q, Lu K, You M, Wang H (2023) Subclinical atherosclerosis in primary Sjögren’s syndrome: comparable risk with diabetes mellitus. Clin Rheumatol 42(6):1607–1614. 10.1007/s10067-023-06538-3 36813944 10.1007/s10067-023-06538-3

[64] Feng R, Xiao X, Wang Y, Huang B, Chen J, Cheng G, Jin Y (2024) Metabolic impact of low dose IL-2 therapy for primary Sjögren’s syndrome in a double-blind, randomized clinical trial. Clin Rheumatol 43(12):3789–3798. 10.1007/s10067-024-07165-2 39482484 10.1007/s10067-024-07165-2 PMC11582071

[65] Ozisler C, Kaplanoglu H (2019) Evaluation of subclinical atherosclerosis by ultrasound radiofrequency data technology in patients with Behçet’s disease. Int J Rheum Dis 22(5):781–788. 10.1111/1756-185X.13579 30985087 10.1111/1756-185X.13579

[66] Alis D, Durmaz ESM, Civcik C, Tutuncu M, Saip S, Kocer N, Islak C, Kizilkilic O (2018) Assessment of the common carotid artery wall stiffness by shear wave elastography in Behcet’s disease. Med Ultrason 20(4):446–452. 10.11152/mu-1565 30534651 10.11152/mu-1565

[67] Yu P, Wang Y, Liu T, Yang L, Li D, Zhu J (2025) Assessment of common carotid artery wall stiffness using shear wave elastography: a promising technique for evaluating active and inactive Behçet’s disease. Quant Imaging Med Surg 15(3):2175–2182. 10.21037/qims-24-1448 40160659 10.21037/qims-24-1448 PMC11948440

[68] Ismail MA, Mounir O, Sedky A, Algahlan HA, Abda EA, Radwan AR, Abozaid HS (2023) Exists a role for serum irisin in Egyptian Behcet’s patients with subclinical atherosclerosis? Clin Rheumatol 42(1):179–186. 10.1007/s10067-022-06368-9 36112245 10.1007/s10067-022-06368-9 PMC9823020

[69] Icli A, Cure MC, Cure E, Arslan S, Unal M, Sakiz D, Ozucan M, Toker A, Turkmen K, Kucuk A (2018) Soluble tumor necrosis factor (TNF)-like weak inducer of apoptosis (Tweak) independently predicts subclinical atherosclerosis in Behcet’s disease. Acta Medica (Hradec Kralove) 61(3):86–92. 10.14712/18059694.2018.123 30543512 10.14712/18059694.2018.123

[70] Merashli M, Bucci T, Arcaro A, Gentile F, Ames PRJ (2023) Subclinical atherosclerosis in Behcet’s disease and its inverse relation to azathioprine use: an updated meta-analysis. Clin Exp Med 23(7):3431–3442. 10.1007/s10238-023-01084-3 37169964 10.1007/s10238-023-01084-3

[71] Mangoni AA, Zinellu A (2024) Circulating cell adhesion molecules in systemic sclerosis: a systematic review and meta-analysis. Front Immunol 15:1438302. 10.3389/fimmu.2024.1438302 39234240 10.3389/fimmu.2024.1438302 PMC11371573

[72] Zinellu A, Mangoni AA (2024) Vascular endothelial growth factor as a potential biomarker in systemic sclerosis: a systematic review and meta-analysis. Front Immunol 15:1442913. 10.3389/fimmu.2024.1442913 39669565 10.3389/fimmu.2024.1442913 PMC11634811

[73] Bai R, Zhang Y, Liu W, Ma C, Chen X, Yang J, Sun D (2019) The relationship of ankylosing spondylitis and subclinical atherosclerosis: a systemic review and meta-analysis. Angiology 70(6):492–500. 10.1177/0003319718814309 30497278 10.1177/0003319718814309

[74] Hintenberger R, Affenzeller B, Vladychuk V, Pieringer H (2023) Cardiovascular risk in axial spondyloarthritis-a systematic review. Clin Rheumatol 42(10):2621–2633. 10.1007/s10067-023-06655-z 37418034 10.1007/s10067-023-06655-z PMC10497445

[75] Masi AT, Fessler SL, Brezka ML, Wang Y, Donohue SE (2023) Systematic review and meta-analysis of individual serum lipids and analysis of lipid ratios in ankylosing spondylitis and healthy control cohorts: significantly lower mean HDL-cholesterol level in ankylosing spondylitis cohorts. Clin Exp Rheumatol 41(9):1862–1874. 10.55563/clinexprheumatol/gtcard 36826790 10.55563/clinexprheumatol/gtcard

[76] Cure E, Icli A, Uslu AU, Sakiz D, Cure MC, Baykara RA, Yavuz F, Arslan S, Kucuk A (2018) Atherogenic index of plasma: a useful marker for subclinical atherosclerosis in ankylosing spondylitis : AIP associate with cIMT in AS. Clin Rheumatol 37(5):1273–1280. 10.1007/s10067-018-4027-0 29435680 10.1007/s10067-018-4027-0

[77] Gasparyan AY, Stavropoulos-Kalinoglou A, Mikhailidis DP, Toms TE, Douglas KM, Kitas GD (2010) The rationale for comparative studies of accelerated atherosclerosis in rheumatic diseases. Curr Vasc Pharmacol 8(4):437–449. 10.2174/157016110791330852 19758114 10.2174/157016110791330852

